# Development of similar materials for fluid–solid coupling model testing and application in damage constitutive models

**DOI:** 10.1038/s41598-024-65242-7

**Published:** 2024-06-26

**Authors:** Xu Ren, Guihong Xu, Ziwei Chen, Shixin Ran, Jie Zhang

**Affiliations:** 1 College of Civil Engineering, Guizhou Institute of Technology, Guiyang, 520025 China; 2 College of Civil Engineering, Guizhou University, Guiyang, 520025 China

**Keywords:** Similar materials, Fluid–solid coupling tests, Sensitivity analysis, Hydraulic properties, Damage constitutive model, Civil engineering, Composites

## Abstract

In order to provide suitable material selection for such fluid–solid coupling model tests, orthogonal experimental studies were conducted using iron concentrate powder and barite powder as aggregates, cement as cementitious materials, and gypsum and clay as modifiers. This research showed: (1) The ATC plays a dominant role in controlling the strength indexes and water absorption of the material, and these indexes show a significant decrease with the increase of the bone adhesive ratio. For each level of ATC increase, the compressive strength decreases by 0.2 MPa, the elastic modulus decreases by 10–20 MPa, and the cohesion decreases by 25–45 kPa. (2) Mixing gypsum and cement cannot jointly promote strength growth. (3) With the increase of GTC, the water absorption rate of the material increases, while the softening coefficient and permeability coefficient decrease obviously. Gypsum, which accounts for 4–16% of cement content, can be suitable for studying the hydraulic properties of similar materials for most sedimentary rock. Based on *Weibull* statistical damage theory, a damage constitutive model for the entire process of rock triaxial compression under the combined action of rainwater infiltration and load was established. Due to the influence of internal pores, the experimental and theoretical results have a certain deviation, the higher the confining pressure, the more obvious the deviation. In addition, the higher the rock strength, the less obvious the deviation caused by pores. This damage model can better describe the progressive failure process of rocks after rainwater infiltration, and can provide theoretical reference for the study of slope stability caused by rainwater infiltration.

## Introduction

Concentrated rainfall often induces geological disasters, mainly due to the infiltration of rainwater, leading to rock mass instability^1,2^. To determine the evolution mechanism and progressive failure law of geological hazards in this type of rock mass, it is necessary to fully consider the physical and mechanical properties of rock and soil particles at the micro-scale and establish a more reasonable mechanical behavior at the macro scale^3^. The geomechanical model test can effectively solve the above problems, and the selection of materials in the experiment is essential. The similarity relationship between the physical and mechanical indexes of the materials is the key to the success of the experiment^4,5^. Some scholars have proposed that indexes such as cohesion $c$ and permeability coefficient $k_{w}$ are the main factors causing model distortion^6^. Especially in fluid–solid coupling experiments, it is more necessary to pay attention to the similarity of hydraulic properties^7^.

At present, scholars have summarized many valuable experiences for the development of similar materials. Shi et al.^7^ conducted experimental research on the changes in the hydraulic properties of materials by adjusting the sand cement ratio, water cement ratio, and particle size of aggregates. Xu Tao. et al.^8^ selected iron powder, putty powder, and quartz sand as aggregates, and cemented them with gypsum to prepare a similar material with a wide range of mechanical parameters. Lei Zhongdai et al.^9^ conducted sensitivity analyses on the density, cohesion, compressive8 strength, and other indexes of similar materials through orthogonal experiments. Ren Xu et al.^10^ found that similar materials bonded with gypsum should be cured for at least 7 days or cured to constant weight. In addition, some scholars have used laundry detergent and hydraulic oil to simulate the viscosity of rocks^11–13^. Currently, most studies focus on the strength index as the primary control factor to develop the mix ratio^11–13^, and there are few systematic studies on the hydraulic properties of similar materials. However, viscous agents such as laundry detergent and hydraulic oil form films in materials, which can restrict the study of hydraulic properties, resulting in limitations in their use in fluid–solid coupling tests. In addition, materials bonded solely with gypsum disintegrate when exposed to water. In contrast, materials bonded solely with cement have no significant softening effect, indicating that similar materials made solely with these two binders are not applicable in fluid–solid coupling tests. It is difficult to obtain similar materials suitable for both soft and hard rock layers in the same type of ratio, which may lead to model distortion due to the lack of continuity of the materials on the contact surface of the soft and hard interlayered rock layers in the model test. In addition, for the development of similar materials, most studies focus on the similarity of physical and mechanical indexes while neglecting the deformation characteristics of materials under stress.

Based on the above reasons, the article conducted orthogonal experimental studies on similar materials using iron powder and barite powder as aggregates, cement as cementitious materials, and gypsum and clay as modifiers. A similar material development plan with comprehensive mechanical parameters suitable for soft and hard rocks, as well as fluid–solid coupling tests has been proposed. Sensitivity analysis was conducted on the density, compressive strength, cohesion, permeability coefficient, softening coefficient, and other indexes of the material. The influence rules of each factor on the above indexes were summarized. And use regression analysis to prepare similar materials for the geomechanical model test of soft and hard rocks of a large-scale landslide treatment project. On this basis, a further study was conducted on the damage constitutive model of progressive failure of rocks after rainwater infiltration. The research results can provide new ideas for the selection of similar materials for fluid–solid coupling tests of soft and hard interbedded rock masses, and also provide necessary theoretical support for the stability problems of rock slope caused by rainwater infiltration.

### Similarity principle

The geomechanical model test is a common method for studying complex engineering geological problems such as slopes and underground engineering^14–16^. The similarity theory is the theoretical foundation of model testing, and the physical model is established between the prototype and the model through the similarity theory, so that the results of model testing can reproduce the actual engineering situation^4,5^. The geometric similarity ratio is assumed to be $C_{l} = n$, and the material is isotropic. In addition, in order to ensure the geomechanical model test in the gravity stress field, the similar ratio of gravitational acceleration is assumed to be 1, that is, $C_{g} = 1$. According to the similarity theory^17,18^, the relevant similarity constants are obtained in Table 1.

**Table 1:** Similarity constant of model experiments.

Physical quantity	Similarity relationship	Similarity constant	Physical quantity	Similarity relationship	Similarity constant
Length	$$C_{l}$$	$$n$$$$n$$	Cohesion	$$C_{c} = C_{\rho } C_{g} C_{l}$$	$$n$$
Gravitational acceleration	$C_{g} = 1$	1	Internal friction angle	$C_{\varphi } = 1$	1
Density	$C_{\rho } = 1$	1	Softening coefficient	$C_{\lambda } = 1$	1
Compressive strength	$C_{\sigma } = C_{\rho } C_{g} C_{l}$	$n$	Permeability coefficient	$C_{k} = \left( {C_{g} C_{l} } \right)^{{{1 \mathord{\left/ {\vphantom {1 2}} \right. \kern-0pt} 2}}}$	$n^{{{1 \mathord{\left/ {\vphantom {1 2}} \right. \kern-0pt} 2}}}$
Elastic modulus	$C_{E} = C_{\rho } C_{l}$	$n$	Water absorption	$C_{w} = 1$	1

### Similar materials

#### Raw materials

**Figure 1:**
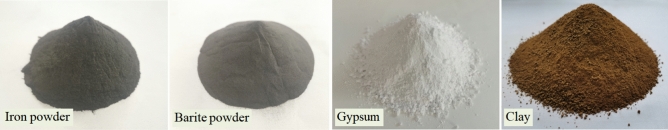
Raw materials.

Select dense, hard iron powder and barite powder as aggregates; Cement is used as a cementitious material. Gypsum is used as a modifier, mainly utilizing its high elasticity and reduced strength when exposed to water after hardening. In addition, adding clay is mainly to ensure that the material has good workability even at low cement content.The raw materials are shown in Fig. 1, and the relevant parameters are shown in Table 2.

**Table 2:** The relevant parameters of raw materials.

Raw materials	Main components	Particle size	Density (g/cm^3^)	Character
Iron powder	iron > 99.5%	16-30mesh	5.40	black particles
Barite powder	barite > 95%	16-30mesh	4.0	black gray particles
Composite portland cement(P.C42.5)	3CaO.SiO_2_, 2CaO.SiO_2_	80μmsquare hole sieve with a residue of no more than 10%	1.28	black gray powder
α Type high-strength gypsum powder	CaSO_4_.1/2H_2_O	200-400mesh	0.90	white powder
Clay (dried)	SiO_2_、Al_2_O_3_	> 10mesh	1.30	yellow particles

#### Orthogonal design

The orthogonal experimental method is a multifactor and multi-level experimental method^19^. It can effectively reduce the number of tests and conveniently obtain test results. Therefore, next, the orthogonal experimental method will be used to conduct experimental research. Four factors are designed here, with the ratio of aggregate to cement (referred to as ATC) as factor A, the ratio of iron powder to barite powder (referred to as ITB) as factor B, the ratio of clay to cement (referred to as CTC) as factor C, and the ratio of gypsum to cement (referred to as GTC) as factor D. To reflect the impact patterns of various factors better, 5 levels were designed for each factor, and the orthogonal design levels for each factor are shown in Table 3.

**Table 3:** Orthogonal design level of various factors.

Level	Factor A	Factor B	Factor C	Factor D
1	15:1	5:5	1.2:1.0	0%
2	16:1	4:6	1.1:1.0	4%
3	17:1	3:7	1.0:1.0	8%
4	18:1	2:8	0.9:1.0	12%
5	19:1	1:9	0.8:1.0	16%

The article adopts a four-factor and five-level orthogonal experimental design L25 (4^5). According to the orthogonal table design experiment, only 25 sets are needed to better represent the results of the comprehensive experiment. According to the orthogonal design level of four factors, the orthogonal experiment ratio scheme for similar materials is shown in Table 4.

**Table 4:** Orthogonal experimental design ratio scheme.

Test number	Factor A	Factor B	Factor C	Factor D
T1	15:1	1:9	1.2:1.0	0%
T2	15:1	5:5	1.1:1.0	4%
T3	15:1	4:6	1.0:1.0	8%
T4	15:1	3:7	0.9:1.0	12%
T5	15:1	2:8	0.8:1.0	16%
T6	16:1	1:9	1.1:1.0	8%
T7	16:1	5:5	1.0:1.0	12%
T8	16:1	4:6	0.9:1.0	16%
T9	16:1	3:7	0.8:1.0	0%
T10	16:1	2:8	1.2:1.0	4%
T11	17:1	1:9	1.0:1.0	16%
T12	17:1	5:5	0.9:1.0	0%
T13	17:1	4:6	0.8:1.0	4%
T14	17:1	3:7	1.2:1.0	8%
T15	17:1	2:8	1.1:1.0	12%
T16	18:1	1:9	0.9:1.0	4%
T17	18:1	5:5	0.8:1.0	8%
T18	18:1	4:6	1.2:1.0	12%
T19	18:1	3:7	1.1:1.0	16%
T20	18:1	2:8	1.0:1.0	0%
T21	19:1	1:9	0.8:1.0	12%
T22	19:1	5:5	1.2:1.0	16%
T23	19:1	4:6	1.1:1.0	0%
T24	19:1	3:7	1.0:1.0	4%
T25	19:1	2:8	0.9:1.0	8%

### Orthogonal experiment results and analysis

#### Experiment results

**Figure 2:**
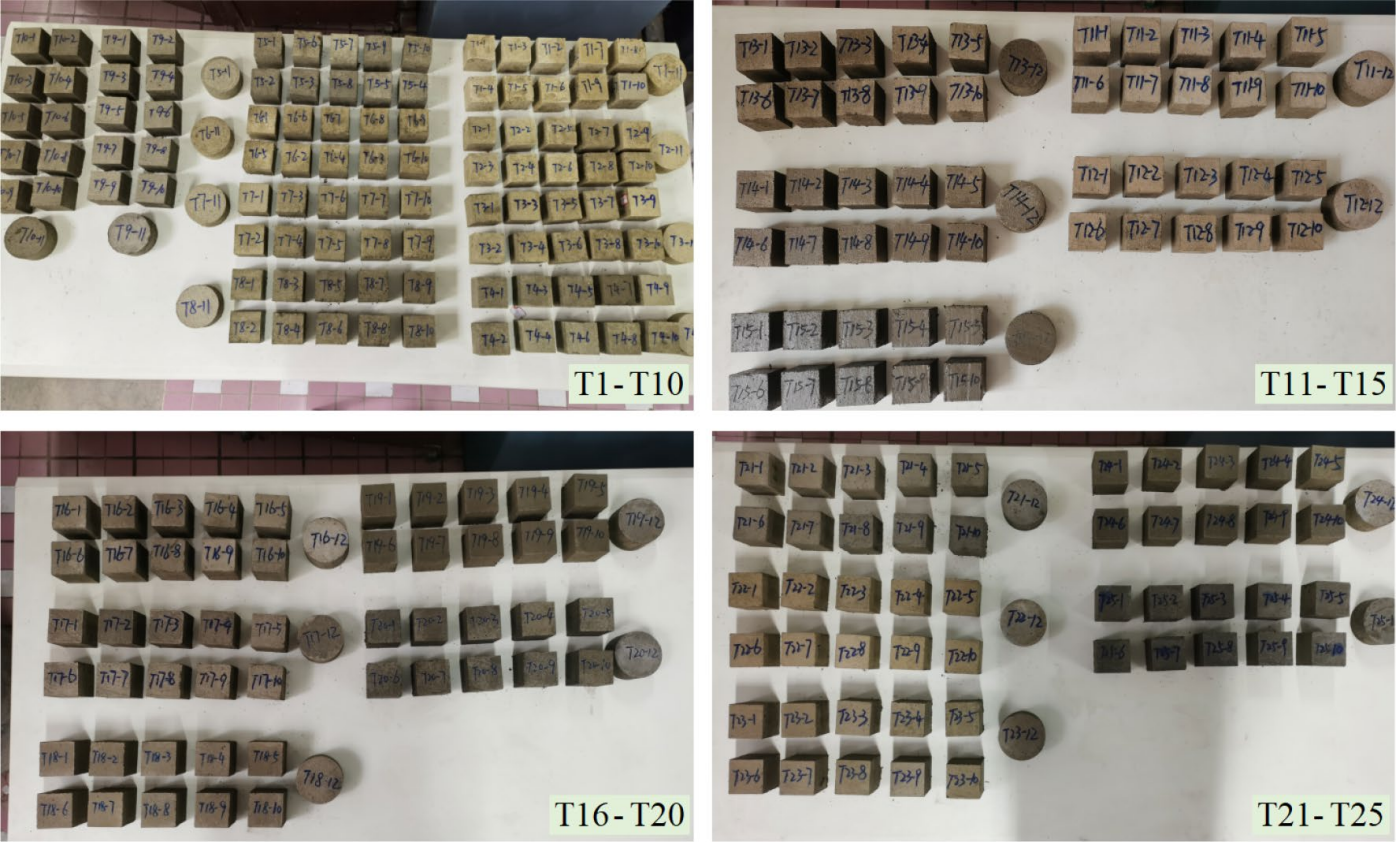
Sample production.

For the above 25 groups of tests, the tests were conducted in batches, as shown in Fig. 2. Maintain for 7 days under dry indoor conditions. After completing the curing, weigh each specimen of the test group and then conduct other physical and mechanical parameters testing.

##### Triaxial test

**Figure 3:**
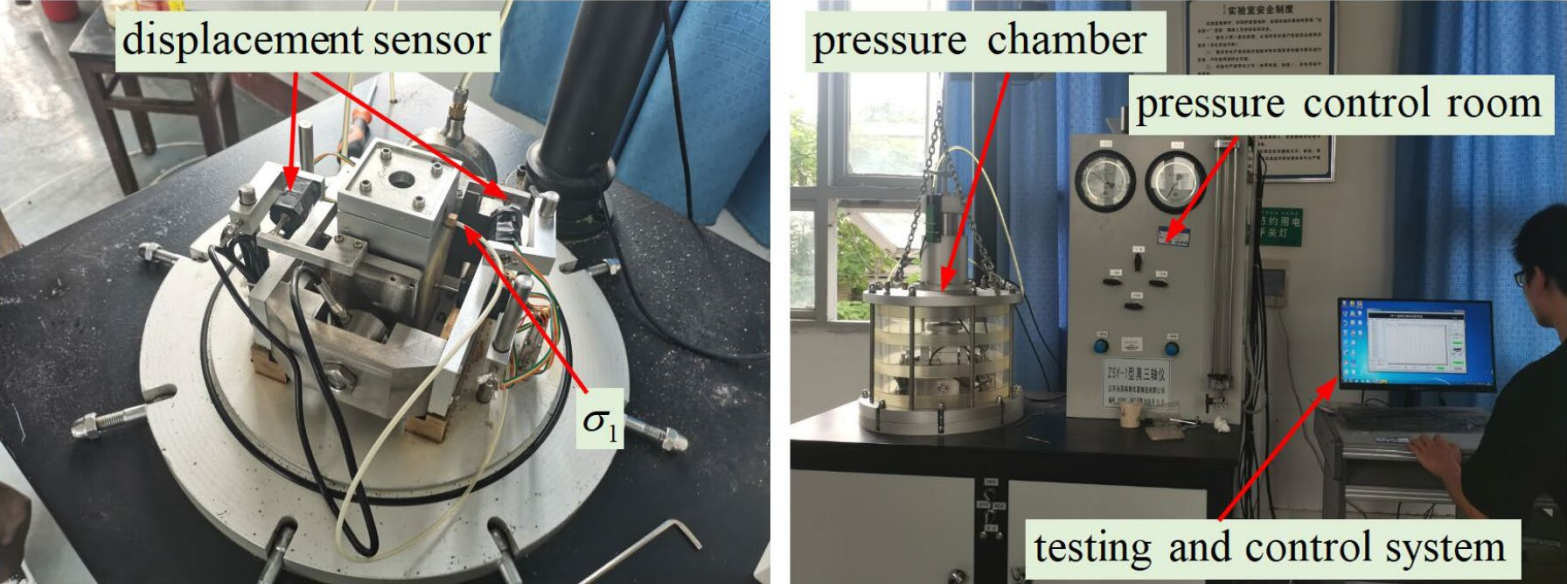
Triaxial test.

Using ZSY-1 true triaxial apparatus for general triaxial testing. Apply confining pressures of 50 kPa, 100 kPa, 200 kPa, and 300 kPa to four specimens in each test group. Load with strain control method at a speed of 0.02 mm/s. The test process is shown in Fig. 3. Based on the experimental results, linear fitting is performed to obtain the Mohr envelope of the group of specimens, and then the shear strength index is obtained.

##### Water absorptivity test

Take 3 specimens from each test group and conduct the test according to the requirements of the "Code for Rock Testing in Highway Engineering" (JTG E41-2018). After water uptake is completed, remove the test piece, use filter paper to absorb surface moisture, and weigh again. The water absorption is expressed in $w$ and calculated using the following formula:

1 $$ w = \frac{{m_{1} - m_{0} }}{{m_{0} }} \times 100\% $$

where $m_{0}$ is the quality before water absorption(g), $m_{1}$ is the quality after water absorption(g).

##### Unconfined compression strength test

**Figure 4:**
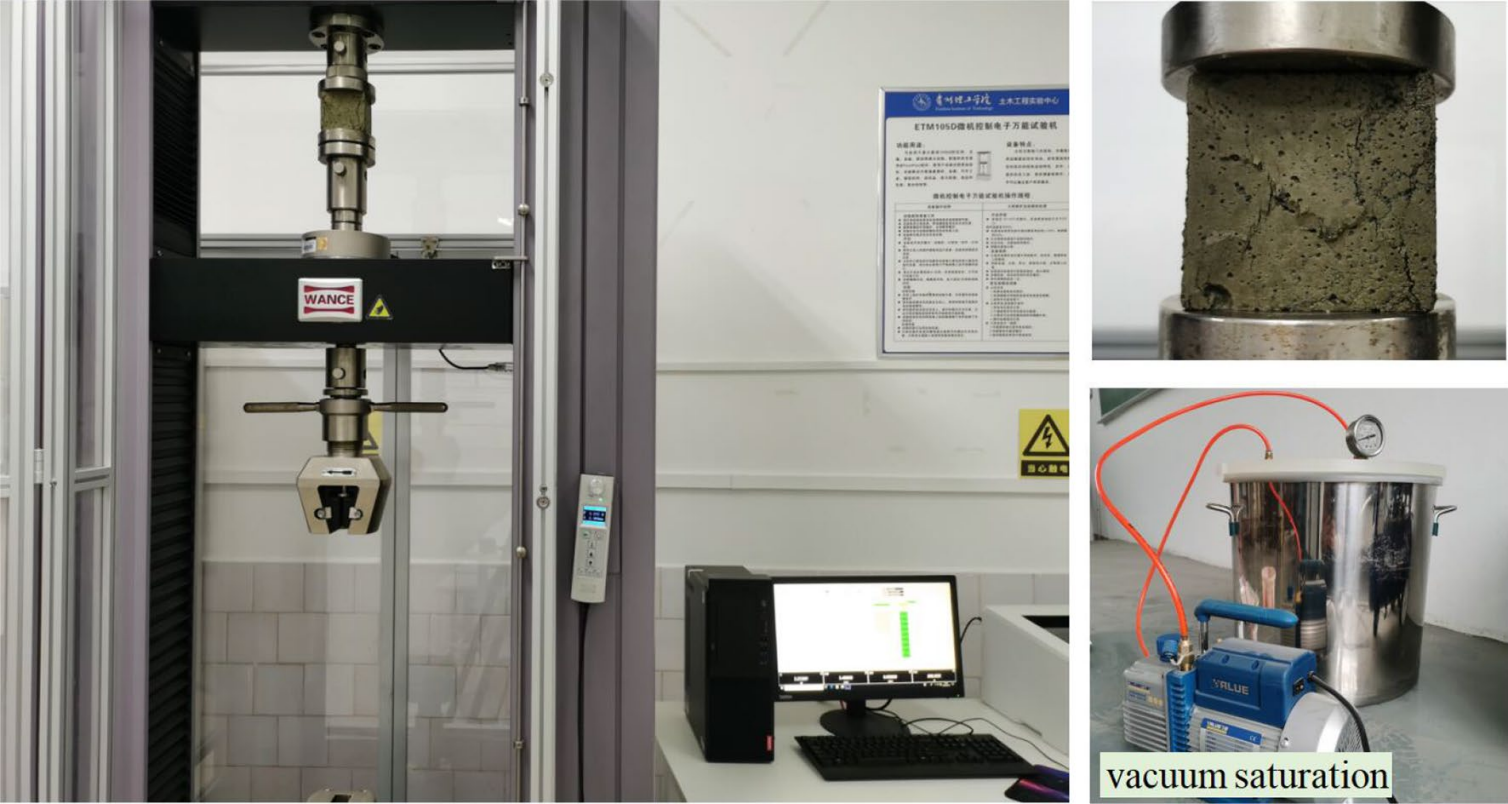
Unconfined compression strength test.

Each group of unconfined compressive strength tests is conducted in two batches. The first batch consists of three specimens that have not been saturated with water (hereafter referred to non-saturated specimens), and the second batch consists of three specimens that have been saturated. Immerse the water absorption test specimen again in a vacuum saturation cylinder, continuously pump until the vacuum is reached, then stop and let it stand for 4 h before taking out the specimen. The test was conducted on a 40kN microcomputer controlled universal testing machine, using a strain controlled loading method at a loading speed of 0.02 mm/min. The test process is shown in Fig. 4. The unconfined compressive strength values of the non-saturated specimens and saturated specimens were obtained through the experiment, and the softening coefficient $\lambda$ was calculated using the following formula:

2 $$ \lambda = \frac{{f_{c} }}{{F_{c} }} $$

where $f_{c}$ is the unconfined compressive strength of specimens after saturation (MPa), $F_{c}$ is the unconfined compressive strength of unsaturated specimens (MPa).

##### Penetration test

**Figure 5:**
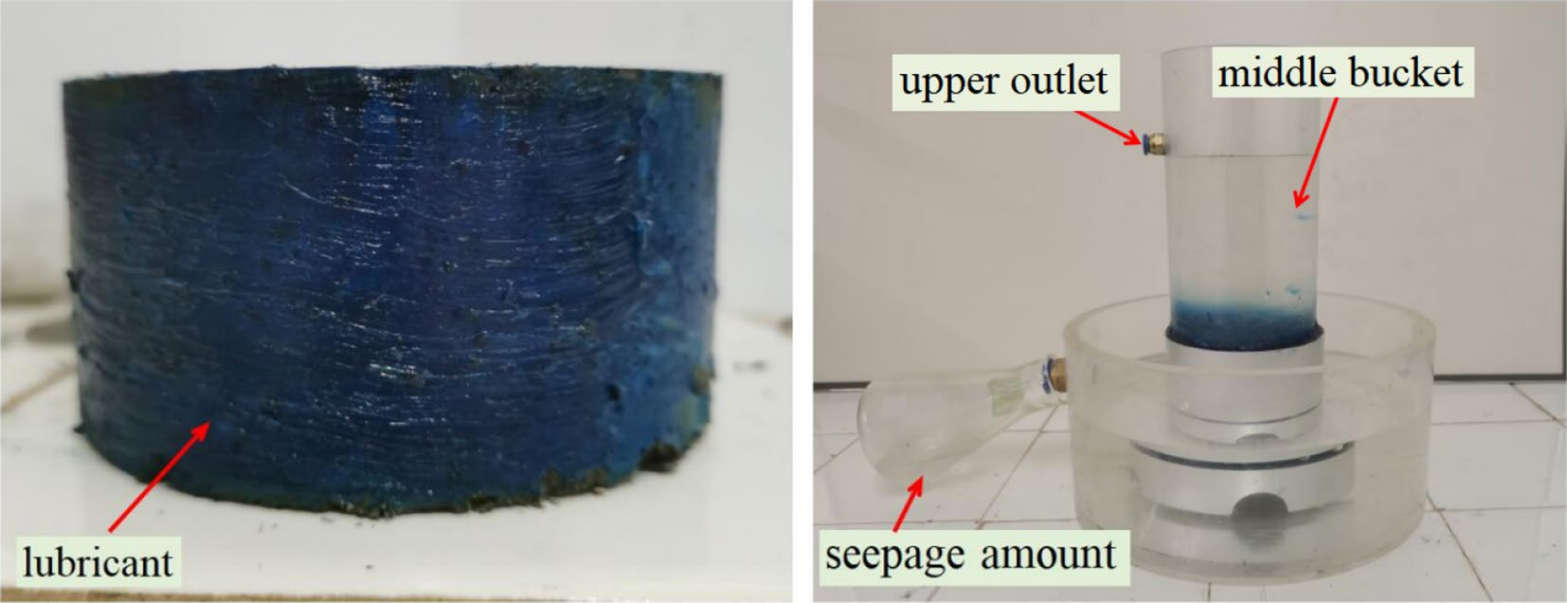
Penetration test.

Apply lubricant(Mobilgrease xhp222, extreme pressure anti-wear high temperature lithium complex grease) on the side of the cylindrical specimen to seal the pores, while ensuring a seamless connection between the test instrument and the side of the specimen, to avoid water leakage from the side wall. The upper limit of adding water in the middle bucket is lower than the upper outlet, so that no water flows out of the upper outlet during the experiment. After the water flow is uniform (about 10 min), measure the water level difference and record the seepage amount for 30 min. The test process is shown in Fig. 5. According to Darcy's law, calculate the permeability coefficient $k_{w}$ through the following formula:

3 $$ k_{w} = \frac{Qd}{{AHt}} $$

where $Q$ is the seepage amount(mm^3^), $d$ is thickness of the specimen(mm), $A$ is the cross-sectional area of the specimen(mm^2^), $H$ is water level difference(mm), $t$ is seepage time(s).

Through the above tests, the test results of 25 sets of specimens are shown in Table 5.

**Table 5:** Orthogonal test results.

Test number	Density (g/cm^3^)	Compressive strength (MPa)	Elastic modulus (MPa)	Cohesion (kPa)	Internal friction angle (°)	Softening coefficient	Water absorption (%)	Permeability coefficient (× 10^-5^ mm/s)
T1	2.571	1.633	160.22	476.7	49.09	0.626	6.04	14.78
T2	2.696	1.714	163.19	442.13	46.12	0.617	6.74	13.16
T3	2.675	1.430	140.63	387.79	45.74	0.590	6.84	6.77
T4	2.578	1.477	134.07	351.89	41.33	0.564	7.45	7.36
T5	2.555	1.130	124.25	343.70	35.72	0.505	8.64	3.28
T6	2.566	1.376	141.21	382.52	44.35	0.621	6.48	10.48
T7	2.716	1.033	135.71	362.37	39.77	0.605	6.90	6.20
T8	2.645	1.177	130.22	305.75	37.50	0.527	7.75	6.29
T9	2.713	1.543	124.39	458.39	37.91	0.584	6.45	5.52
T10	2.600	1.333	126.53	352.46	43.12	0.624	4.70	15.44
T11	2.576	0.899	135.01	312.66	40.26	0.526	7.30	7.32
T12	2.786	0.980	111.07	348.06	35.77	0.630	4.79	8.82
T13	2.729	1.226	100.88	405.32	37.08	0.642	5.97	8.78
T14	2.679	1.164	132.0	347.31	39.02	0.653	4.97	12.85
T15	2.682	1.181	127.19	330.57	35.66	0.594	6.07	7.92
T16	2.672	0.843	99.08	323.63	35.78	0.659	5.16	6.45
T17	2.762	0.861	90.0	301.16	30.56	0.60	5.56	6.86
T18	2.738	0.977	125.94	318.81	38.42	0.572	6.25	10.30
T19	2.706	0.764	129.49	258.57	33.56	0.624	5.16	9.06
T20	2.670	1.076	101.25	368.48	37.54	0.703	4.71	14.27
T21	2.624	0.658	85.24	225.91	26.99	0.661	4.79	7.56
T22	2.799	0.692	113.34	198.15	30.52	0.642	5.27	9.34
T23	2.767	0.736	58.19	314.87	37.64	0.655	4.88	11.79
T24	2.749	0.666	72.72	296.71	30.35	0.623	4.76	9.0
T25	2.707	0.721	79.68	310.82	29.62	0.632	5.71	6.99

#### Sensitivity analysis

##### Density (ρ)

Calculate the average of the density results obtained from 5 levels of the 4 factors in the orthogonal experiment, and perform range analysis and variance analysis on this basis. The results are shown in Table 6. It can be seen that the degree of impact on density is B-A-D-C from large to small. Among them, factors A and B have a significant impact on density and play a dominant role, while the influence of factor C is very small.

**Table 6:** Range analysis and variance analysis of density (g/cm^3^).

Level	Factor A	Factor B	Factor C	Factor D
1	2.615	2.752	2.677	2.701
2	2.648	2.711	2.683	2.689
3	2.690	2.685	2.677	2.678
4	2.710	2.643	2.678	2.668
5	2.729	2.602	2.677	2.656
Range	0.1142	0.15	0.0068	0.0452
Variance	1.73 × 10^–3^	2.72 × 10^–3^	0.006 × 10^–3^	0.25 × 10^–3^

**Figure 6:**
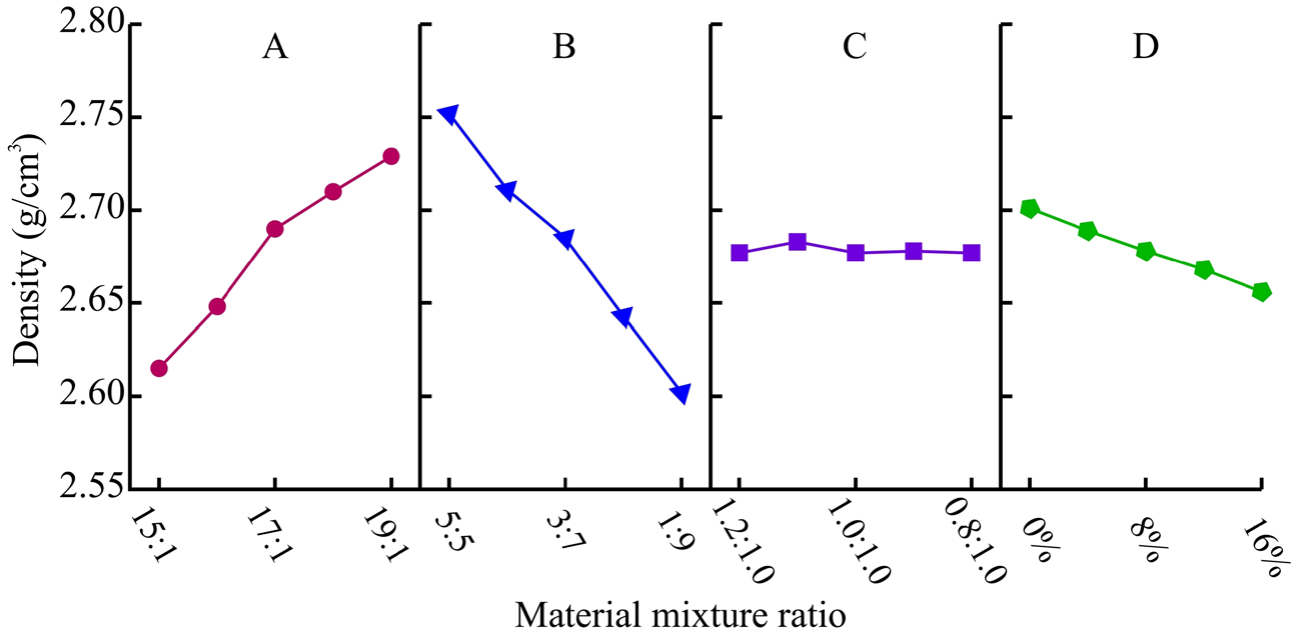
Analysis of sensitive factors for density.

Based on the results in Table 6, a visual analysis diagram of the impact of various factors on density is obtained, as shown in Fig. 6. It can be found that an increase in ITB will inevitably increase the density of the material, and an increase in ATC will also have the same effect. In addition, the low-density characteristics of gypsum also have a certain impact on this index, while the amount of clay does not affect the density of the material. Overall, the change in density is not significant, mainly due to the small difference in density between the two aggregates.

##### Compressive strength (F_c_)

**Figure 7:**
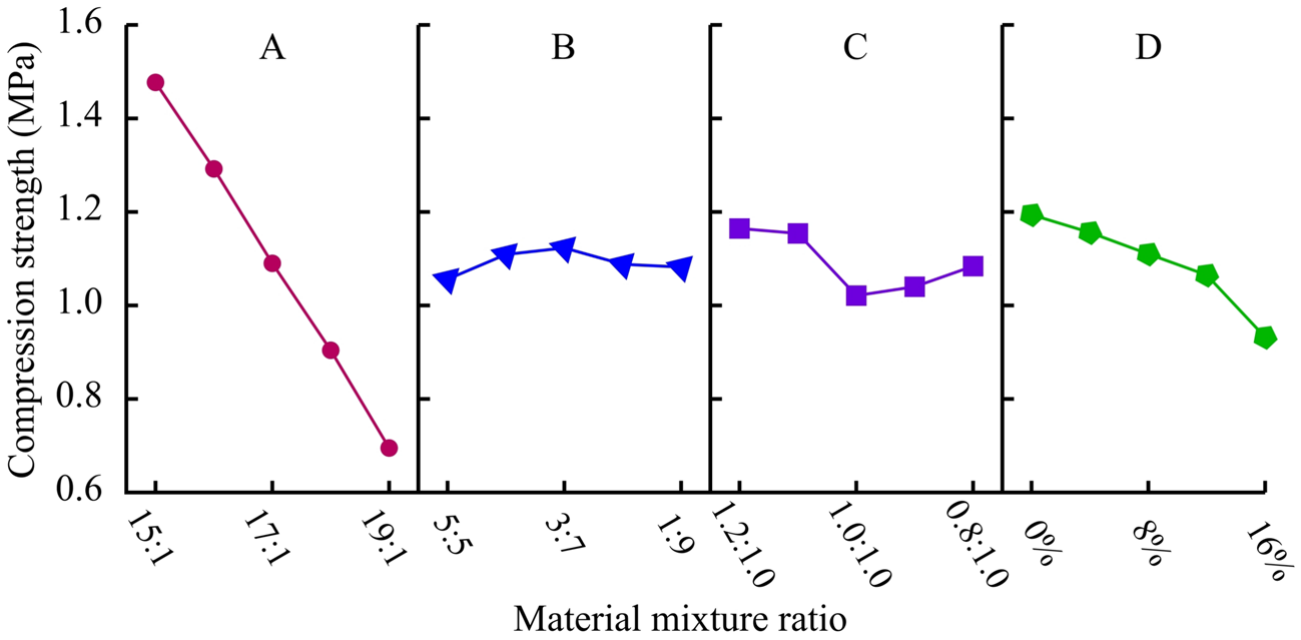
Analysis of sensitive factors for compressive strength.

**Figure 8:**
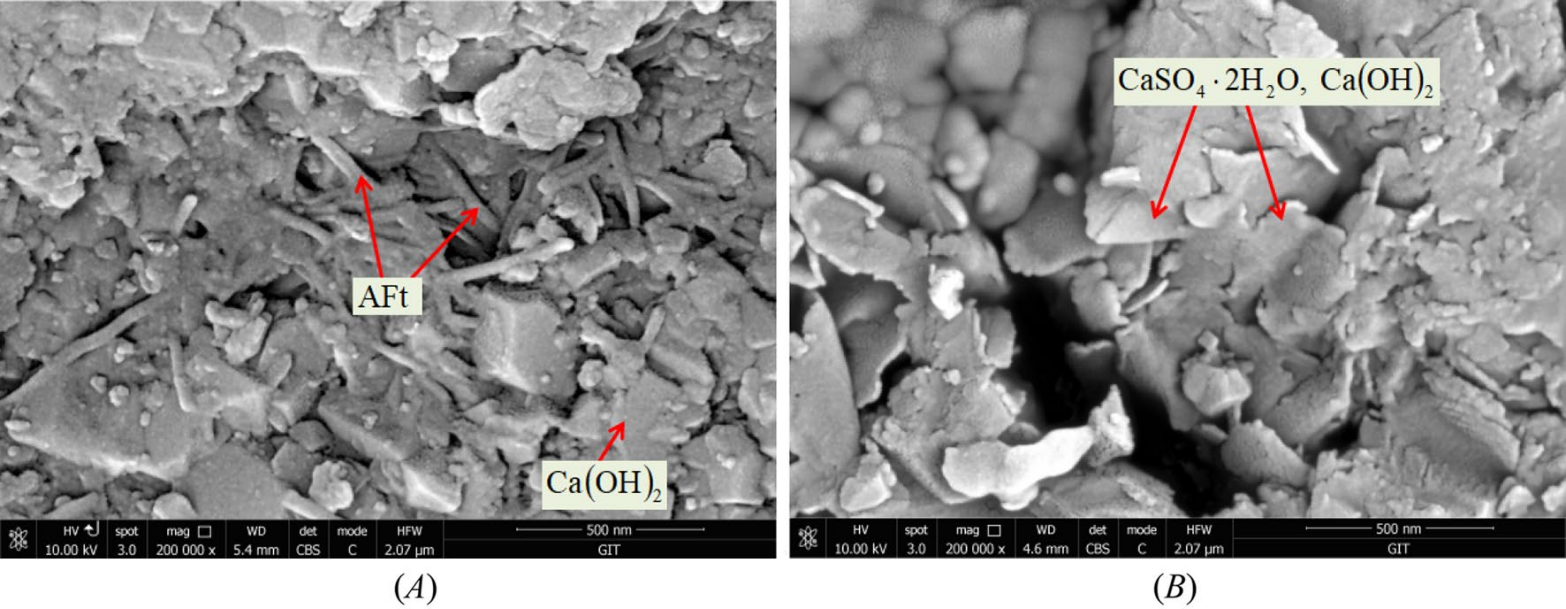
200,000 times XRD images of hydration products.

Use the same method for range analysis and variance analysis. From the results in Table 7, it can be seen that the degree of influence of the four factors on compressive strength varies from large to small(A-D-C-B). It indicates that factor A plays a major regulatory role in the compressive strength of materials, while factor B has a very small impact. Figure 7 is a visual analysis of the impact of four factors on compressive strength. The compressive strength of the material significantly decreases with the increase of ATC, with a maximum decrease of 47.8%. For each additional level of ATC, the compressive strength decreases by 0.2 MPa. When ATC is under low-level conditions, the amount of clay used has no significant impact on the strength. Furthermore, it is worth noting that although the amount of gypsum powder used is relatively small, it also has a significant regulatory effect on strength. The compressive strength decreases by approximately 0.04 MPa for every 4% increase in gypsum content. After adding gypsum powder, the amount of ettringite in the hydration products decreases. Figure 8 shows a 200,000 times XRD image maintained for 7 days. “Needle-shapes” AFt crystals can be observed in a network connection in Fig. 8(A) and “Cloud-shapes” Ca (SO_4_)·2H_2_O and Ca (OH)_2_ crystals can be observed in Fig. 8(B). It can be seen that a small amount of gypsum plays an obvious restricting role in the hydration reaction of cement. The above is the reason for the decrease in strength. Therefore, the mixing of gypsum and cement does not jointly promote the development of material strength, but rather has a clear opposite effect.

**Table 7:** Range analysis and variance analysis of compressive strength (MPa).

Level	Factor A	Factor B	Factor C	Factor D
1	1.477	1.056	1.160	1.194
2	1.292	1.109	1.154	1.156
3	1.090	1.123	1.021	1.110
4	0.904	1.088	1.040	1.065
5	0.695	1.082	1.084	0.932
Range	0.782	0.067	0.076	0.261
Variance	76.29 × 10^–3^	0.53 × 10^–3^	3.27 × 10^–3^	8.20 × 10^–3^

##### Elastic modulus (E)

**Figure 9:**
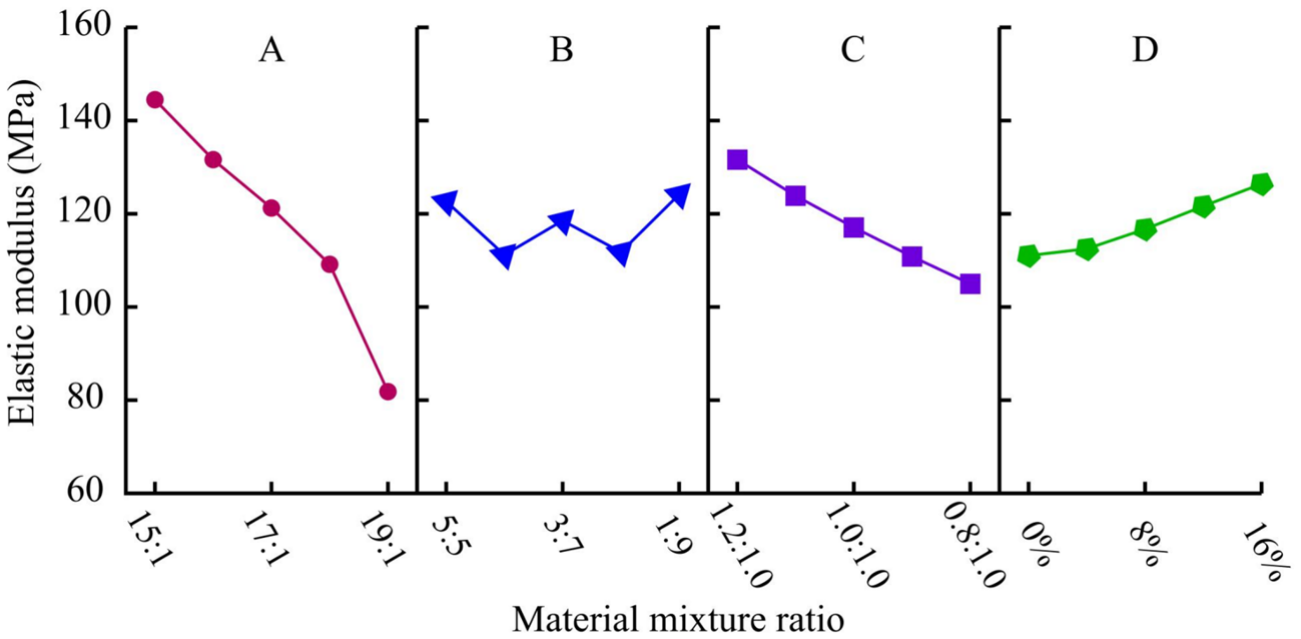
Analysis of sensitive factors for elastic modulus.

The range analysis and variance analysis of elastic modulus results are shown in Table 8. The sensitivity of the four factors to elastic modulus is A-C-D-B from large to small, with factor A being significant. Figure 9 is a visual analysis of the impact of four factors on elastic modulus. ATC is the main factor controlling the elastic modulus, which significantly decreases with the increase of ATC. For each level of ATC increase, the elastic modulus decreases by 10-20 MPa. In addition, as the clay content decreases, the elastic modulus also shows a significant linear decrease trend. Gypsum also exhibits its high elastic modulus characteristics, and the elastic modulus increases linearly with the increase of it. Additionally, it was found from the experimental results that gypsum has a more significant effect on improving the elastic modulus at high ATC levels.

**Table 8:** Range analysis and variance analysis of elastic modulus (MPa).

Level	Factor A	Factor B	Factor C	Factor D
1	144.47	122.66	131.61	111.02
2	131.61	111.17	123.85	112.48
3	121.23	118.53	117.06	116.70
4	109.15	111.78	110.82	121.63
5	81.83	124.15	104.95	126.46
Range	62.64	12.98	26.65	15.44
Variance	456.43	28.92	88.29	33.00

##### Cohesion (*c*)

**Figure 10:**
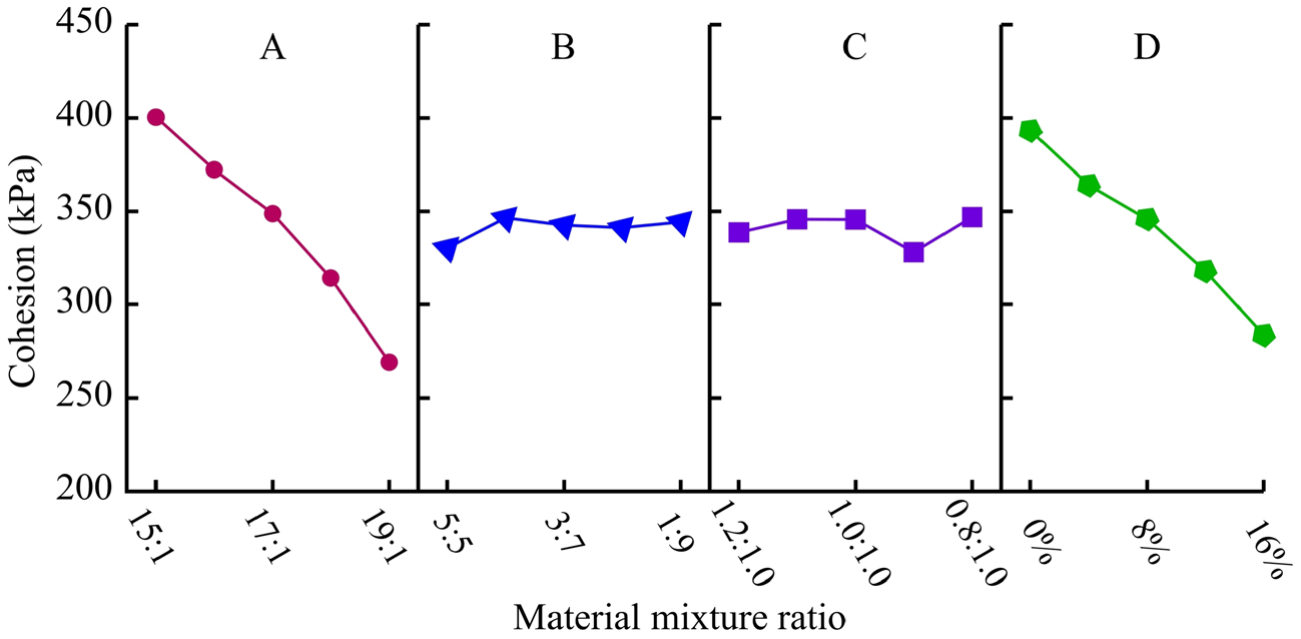
Analysis of sensitive factors for cohesion.

The range analysis and variance analysis of cohesion results are shown in Table 9. Comparing the effects of various factors on cohesion, the sensitivity is ranked in descending order as A-D-C-B. Among them, factors A and D have a significant impact on cohesion, while factors B and C have a very small impact. Figure 10 is a visual analysis of the impact of four factors on cohesion. The cohesion decreases significantly with the increase of ATC. For each level of ATC increase, the cohesion decreases by 25-45 kPa, and the larger the ATC, the more significant the decrease rate. A small amount of gypsum also significantly affects the cohesion, which decreases linearly with the increase of gypsum content. Additionally, the amount of clay used hardly affects the cohesion of the material.

**Table 9:** Range analysis and variance analysis of cohesion (kPa).

Level	Factor A	Factor B	Factor C	Factor D
1	400.44	330.37	338.69	393.30
2	372.30	346.51	345.73	364.05
3	348.78	342.57	345.60	345.92
4	314.13	341.21	328.03	317.91
5	269.29	344.28	346.90	283.77
Range	131.15	16.13	18.87	109.53
Variance	2087.51	31.31	50.38	1419.94

##### Internal friction angle (*φ*)

**Figure 11:**
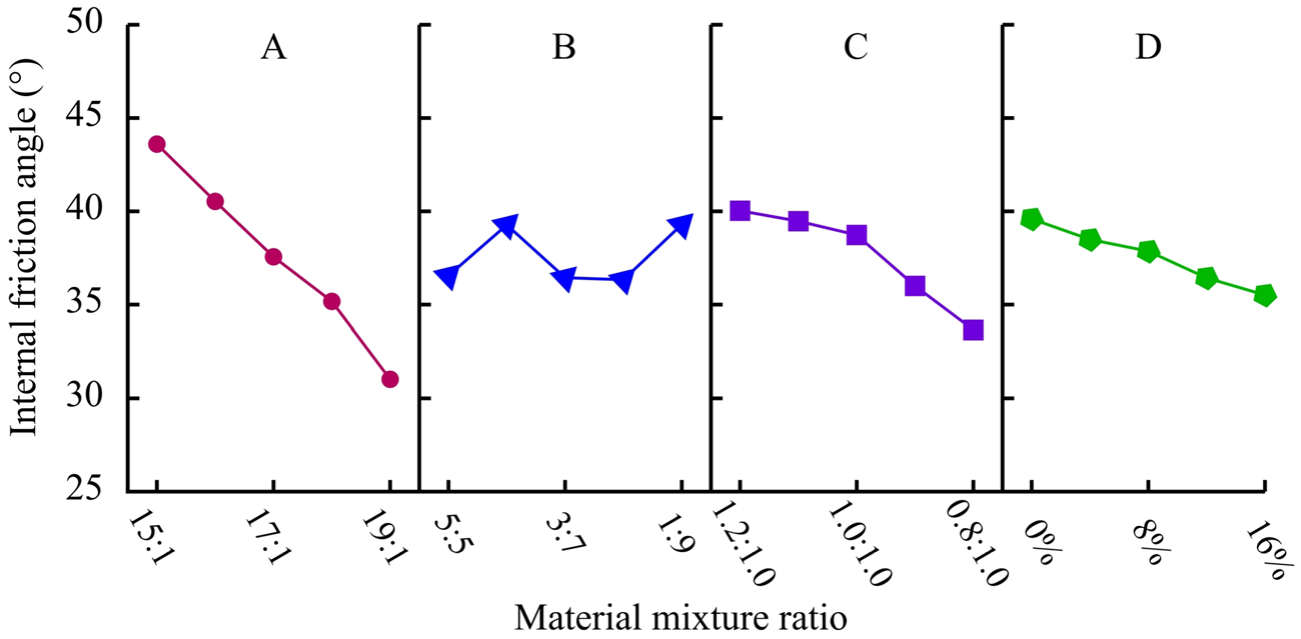
Analysis of sensitive factors for internal friction angle.

The range analysis and variance analysis of internal friction angle results are shown in Table 10. For the internal friction angle of materials, the sensitivity of each factor is ranked from highest to lowest in A-C-D-B, with factor A being the most significant, followed by factor C. Based on the analysis results in Table 10, a visual analysis diagram of the impact of four factors on the internal friction angle is obtained, as shown in Fig. 11. With the increase of ATC and GTC, the internal friction angle of the material decreases linearly, especially when the influence of the former is more significant. In addition, clay can effectively enhance the friction effect between internal particles, resulting in an increase in the internal friction angle of the material. From this, it can be found that in addition to ATC, clay, and gypsum can also be important raw materials for adjusting the internal friction angle.

**Table 10:** Range analysis and variance analysis of internal friction angle (°).

Level	Factor A	Factor B	Factor C	Factor D
1	43.60	36.55	40.03	39.59
2	40.53	39.27	39.47	38.49
3	37.56	36.44	38.73	37.86
4	35.17	36.33	36.0	36.43
5	31.02	39.29	33.65	35.51
Range	12.58	2.96	6.38	4.08
Variance	18.74	1.95	5.77	2.11

##### Softening coefficient (*λ*)

**Figure 12:**
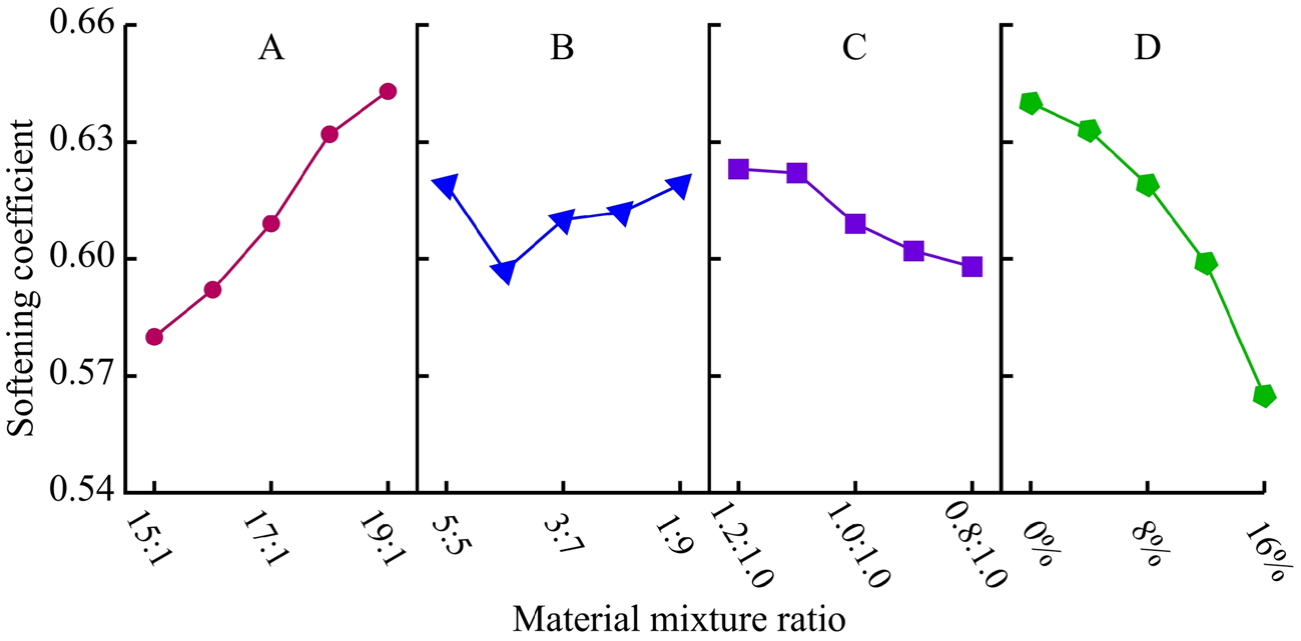
Analysis of sensitive factors for softening coefficient.

The range analysis and variance analysis of softening coefficient results are shown in Table 11. Comparing the effects of various factors on the softening coefficient, the sensitivity is ranked in descending order as D-A-C-B. Among them, the factor D is the most significant, followed by the factor A. Based on the analysis results in Table 11, a visual analysis diagram of the influence of four factors on the softening coefficient is obtained, as shown in Fig. 12. It can be seen that the softening coefficient significantly decreases with the increase of gypsum content, and this effect becomes more apparent with the increase of gypsum content. Due to the formation of Ca (SO_4_)·2H_2_O after gypsum condensation, the dissolution of Ca (SO_4_)·2H_2_O when encountering water causes a certain degree of damage to the material's skeleton, resulting in a significant decrease in strength. It can be concluded that gypsum powder can effectively adjust the softening coefficient of the material, and a gypsum content range of 4% -16% can be applied to most sedimentary rocks, which is of great significance in fluid–solid coupling tests. In addition, the larger the ATC, the higher the strength of the material, but the more significant the decrease in material strength after saturation.

**Table 11:** Range analysis and variance analysis of softening coefficient.

Level	Factor A	Factor B	Factor C	Factor D
1	0.580	0.619	0.623	0.640
2	0.592	0.597	0.622	0.633
3	0.609	0.610	0.609	0.619
4	0.632	0.612	0.602	0.599
5	0.643	0.619	0.598	0.565
Range	0.062	0.021	0.025	0.075
Variance	5.4 × 10^–4^	0.6 × 10^–4^	1.0 × 10^–4^	7.3 × 10^–4^

##### Water absorption (w)

**Figure 13:**
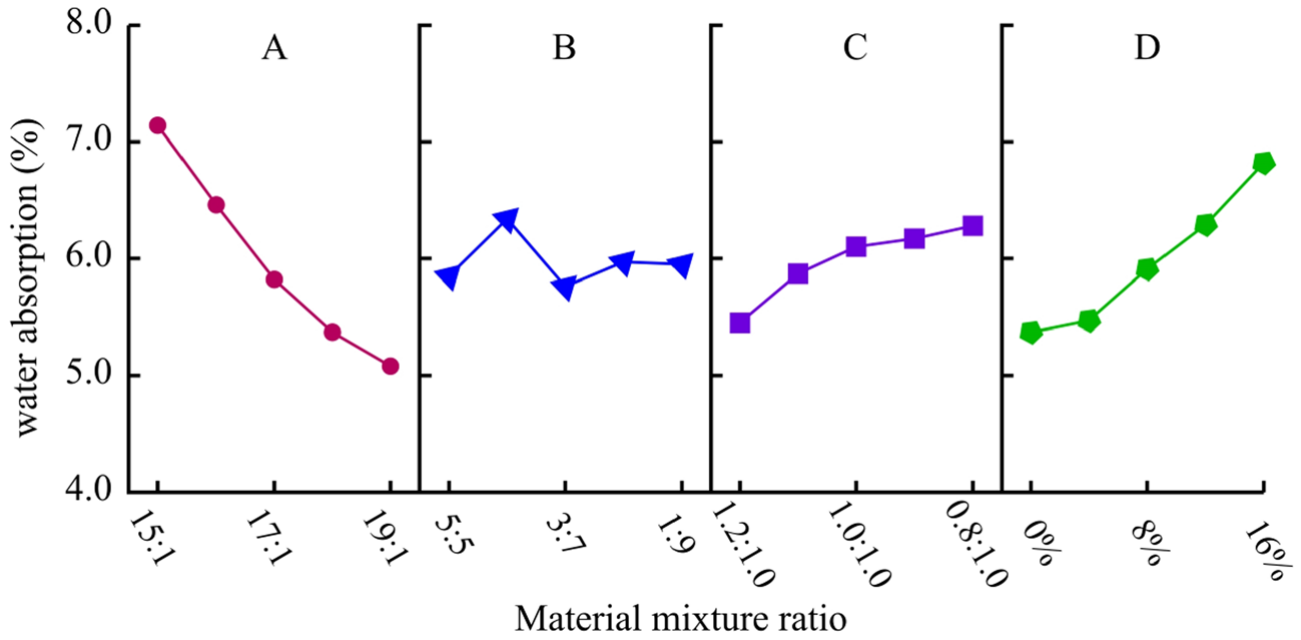
Analysis of sensitive factors for water absorption.

The range analysis and variance analysis results of water absorption are shown in Table 12. It can be seen that the degree of influence on water absorption is A-D-C-B in descending order. Among them, the influence of factors A and D is more significant than other factors. Based on the analysis results in Table 12, a visual analysis diagram of the impact of four factors on water absorption is obtained, as shown in Fig. 13. Overall, the water absorption rate increases with the decrease of ATC. The smaller the ATC, the greater the proportion of cement and the more pores formed after hydration, resulting in an increase in water absorption. The smaller the ATC, the more pores formed after the hydration of the cementitious material, leading to an increase in water absorption. When the gypsum content exceeds 4%, the water absorption increases by 0.3% ~ 0.5% for each additional level. In addition to the porous hydration products, the dissolution of Ca (SO_4_) · 2H_2_O is also one of the reasons for the increase in water absorption.

**Table 12:** Range analysis and variance analysis of water absorption (%).

Level	Factor A	Factor B	Factor C	Factor D
1	7.14	5.85	5.45	5.37
2	6.46	6.34	5.87	5.47
3	5.82	5.76	6.10	5.91
4	5.37	5.97	6.17	6.29
5	5.08	5.95	6.28	6.82
Range	2.06	0.58	0.84	1.45
Variance	0.624	0.107	0.156	0.357

##### Permeability coefficient (k_w_)

**Figure 14:**
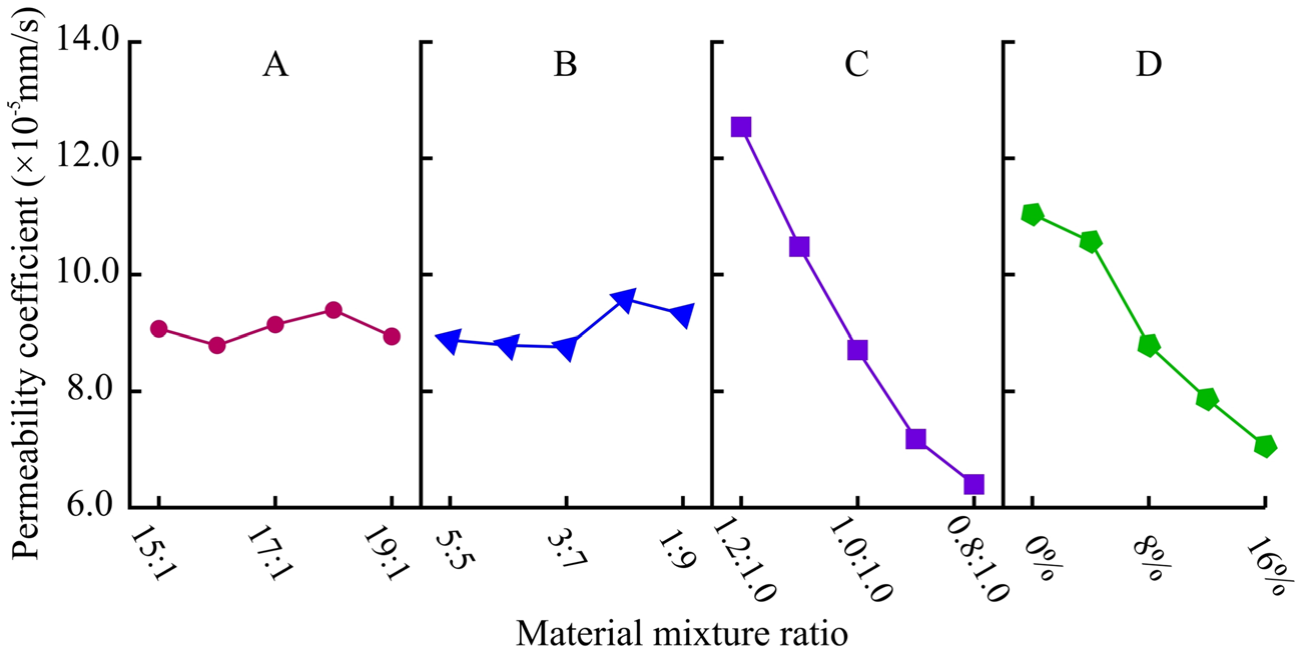
Analysis of sensitive factors for permeability coefficient.

The range analysis and variance analysis results of the permeability coefficient are shown in Table 13. Comparing the effects of various factors on the permeability coefficient, the sensitivity is ranked in descending order as C-D-B-A. Among them, factor C and factor D are the main factors that affect the permeability coefficient. The intuitive analysis diagram is shown in Fig. 14. The permeability coefficient of the material decreases with the decrease of CTC. With the increase of GTC, the permeability coefficient also significantly decreases. However, ATC and ITB have little effect on the permeability coefficient of the material.

**Table 13:** Range analysis and variance analysis of permeability coefficient (× 10^-5^ mm/s).

Level	Factor A	Factor B	Factor C	Factor D
1	9.07	8.88	12.54	11.04
2	8.79	8.79	10.48	10.57
3	9.14	8.76	8.71	8.79
4	9.39	9.58	7.18	7.87
5	8.94	9.32	6.40	7.06
Range	0.60	0.82	6.14	3.98
Variance	0.04	0.11	4.97	2.33

## Material preparation and application

### Material preparation

#### Project overview

**Figure 15:**
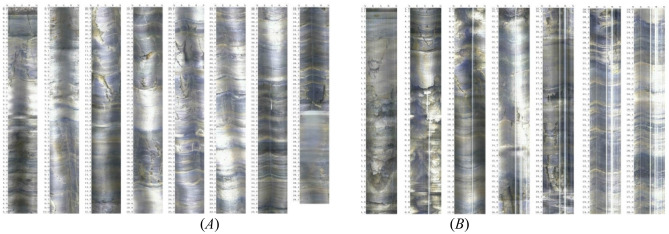
The television images of “muddy limestone with mudstone”.

The slope of a certain ring road in Guiyang City, Guizhou Province is a steep internal bedding slope, with a monoclinic rock formation and a rock occurrence of ∠15°-∠35°. The structural surface is straight and smooth, with intercalated layers containing mud and poor local bonding, making it a weak structural surface. The content of mudstone interlayer accounts for 16% of the total rock layer thickness, with a thickness ranging from 5 to 30 cm, while the rest are argillaceous limestone. This type of rock formation is called "muddy limestone with mudstone" , it is shown in Fig. 15. Collect 16 natural samples of mudstone and muddy limestone to obtain their physical and mechanical indexes. In the geomechanical model test of slope engineering, for the convenience of implementation and economic requirements, the geometric dimensions are generally designed to be 1.0 ~ 2.0 m. Based on the actual engineering situation, the geometric similarity constant is determined to be 35. According to the similarity constants in Table 14, the physical and mechanical indexes of similar materials for mudstone and muddy limestone are obtained, as shown in Table 14.

**Table 14:** Physical and mechanical indexes related to mudstone and muddy limestone.

Indexes	Density ρ (g/cm^3^)	Compressive strength F_c_ (MPa)	Elastic modulus E (MPa)	Cohesion *c* (kPa)	Internal friction angle *φ* (°)	Softening coefficient *λ*	Water absorption w (%)	Permeability coefficient $k_{w}$ (× 10^-5^ mm/s)
Mudstone	2.41–2.63	10.0–25.5	1300–3100	7370–11,050	30.9–38.6	0.46–0.56	3.9 ~ 4.6	0–100
Similar material of mudstone	2.41–2.63	0.33–0.72	37.1–88.6	210.6–315.7	30.9–38.6	0.46–0.56	3.9 ~ 4.6	0–16.9
Muddy limestone	2.65–2.80	34.3–58.7	4900–10,700	9230–14,510	35.0–48.0	0.55–0.67	1.2–3.0	0–60
Similar material of Muddy limestone	2.65–2.80	0.98–1.68	140.0–305.7	263.7–414.6	35.0–48.0	0.55–0.67	1.2–3.0	0–10.1

#### Preparation of similar materials

The water absorption of rocks can effectively reflect the development degree of rock microcracks and can be used to determine the frost resistance and weathering resistance of rocks^20^. The geographical location of the project does not have the impact of frost resistance, so the water absorption index of the rock is ignored here. Regression analysis is an effective method for analyzing the relationship between dependent and independent variables. By conducting regression analysis on the above 25 sets of test results, ignoring some secondary influencing factors, and combining with the requirements for relevant indexes in Table 14, the ratio of similar materials for mudstone and muddy limestone was obtained, as shown in Table 15.

**Table 15:** The ratio of similar materials between mudstone and muddy limestone.

Rock category	Factor A	Factor B	Factor C	Factor D
Similar material of mudstone	18:1	1:9	0.5:1.0	20%
Similar material of muddy limestone	15:1	5:5	1.2:1.0	8%

In regression analysis, it was found that the elastic modulus of similar mudstone materials in the above mix proportions is slightly higher than the requirements in Table 14. Based on the regulatory effect of re-dispersible latex powder on elastic modulus in the reference^10^, a re-dispersible latex powder with a cement content of 6% was added to the mudstone ratio to obtain the final ratio.

### Damage constitutive model of mudstone and muddy after rainwater infiltration

In areas with high rainfall and concentrated rainfall, landslide disasters often occur due to rainwater infiltration. At present, there are many mature research results on the damage constitutive model of rocks under the influence of various factors^21–25^. However, there are few studies on the microstructural damage caused by progressive failure of rocks under external loads after rainwater infiltration. Therefore, exploring the damage constitutive model of rocks after rainwater infiltration is of great significance for in-depth study of the progressive failure evolution law of geological bodies under rainfall conditions.

#### Constitutive model of damage after rainwater infiltration

The infiltration of rainwater leads to changes in the microstructure of the rock, and micro cracks within the rock gradually begin to appear, which is bound to cause irreversible damage to the rock. The changes in microstructure will inevitably affect the physical and mechanical properties of rock masses, and damage mechanics methods can be used to study the damage variables of rainwater infiltration into rock masses^26,27^.

Define the damage variable of rainwater infiltration based on the unconfined compressive strength of rocks, denoted by $D_{w}$, which is defined as follows:

4 $$ D_{w} = 1 - \frac{{f_{c} }}{{F_{c} }} $$

Substitute Eq. (2) into Eq. (4), so $D_{w}$ can be expressed as:

5 $$ D_{w} = 1 - \lambda $$

In addition, the damage to the rock microstructure caused by external loads is denoted by $D_{L}$, and this damage is randomly distributed^28,29^. Assuming that the micro element strain $\varepsilon$ of the rock mass obeys the *Weibull* function distribution, so $D_{L}$ is defined as follows:

6 $$ D_{L} = 1 - \exp \left[ { - \left( {\varepsilon /k} \right)^{m} } \right] $$

where the $m$ and $k$ are distribution parameters.

According to the calculation method of damage under the joint action of two factors proposed by Huang Z. et al.^30^ and Liu Z.X. et al.^31^, the total damage variable $D_{t}$ is obtained, and calculated using the following formula:

7 $$ D_{t} = D_{w} + D_{L} - D_{w} D_{L} $$

Substitute Eqs. (5) and (6) into (7), $D_{t}$ can be expressed as:

8 $$ D_{t} = 1 - \lambda \exp \left[ { - \left( {\varepsilon /k} \right)^{m} } \right] $$

Due to the influence of rainwater infiltration, when the rock is damaged, the external load is borne jointly by the damaged and undamaged parts of the rock. Assuming that the damaged element is loaded by residual stress, there is

9 $$ \sigma_{1} = \sigma_{1}^{*} \left( {1 - D_{t} } \right) + \sigma_{r} D_{t} $$

where $\sigma_{1}$ is nominal stress, $\sigma_{1}^{*}$ is effective stress of the undamaged part, $\sigma_{r}$ is residual stress.

According to the generalized Hooke's theorem, $\sigma_{1}^{*}$ can be expressed as:

10 $$ \sigma_{1}^{*} = E\varepsilon_{1} + \mu \left( {\sigma_{2} + \sigma_{3} } \right) $$

where: $E$ and $\mu$ are the Elastic modulus and Poisson's ratio of rocks (non-saturated state) , $\varepsilon_{1}$ is the nominal strain, $\sigma_{2}$ and $\sigma_{3}$ are the confining pressure.

In general triaxial testing, $\sigma_{2} = \sigma_{3}$. Substitute Eq. (10) into Eq. (9), so the $\sigma_{1}$ can be expressed as:

11 $$ \sigma_{1} = E\varepsilon_{1} \left( {1 - D_{t} } \right) + 2\mu \sigma_{3} \left( {1 - D_{t} } \right) - \sigma_{r} D_{t} $$

Substitute Eq. (8) into (11), the statistical damage constitutive model of rocks after rainwater infiltration was obtained, calculated using the following formula:

12 $$ \sigma_{1} = \left( {E\varepsilon_{1} + 2\mu \sigma_{3} - \sigma_{r} } \right)\lambda \exp \left[ { - \left( {{\varepsilon \mathord{\left/ {\vphantom {\varepsilon k}} \right. \kern-0pt} k}} \right)^{m} } \right] + \sigma_{r} $$

In this model, there is $\sigma_{r} = 0$ before the peak stress. The strain on the microelement is $\varepsilon = \varepsilon_{1} - {{\left( {1 - 2\mu } \right)\sigma_{3} } \mathord{\left/ {\vphantom {{\left( {1 - 2\mu } \right)\sigma_{3} } E}} \right. \kern-0pt} E}$. The distribution parameters $m$ and $k$ are determined by the peak point $\left( {\sigma_{p} ,\varepsilon_{p} } \right)$ of the rock stress–strain curve. When $\varepsilon_{1} = \varepsilon_{P}$, $\sigma_{1} = \sigma_{P}$; when $\varepsilon_{1} = \varepsilon_{P}$, ${{d\sigma_{1} } \mathord{\left/ {\vphantom {{d\sigma_{1} } {d\varepsilon_{1} }}} \right. \kern-0pt} {d\varepsilon_{1} }} = 0$. Combining geometric control equations, the $m$ and $k$ are as follows:

13 $$ m = \frac{{E\varepsilon_{p} - \left( {1 - 2\mu } \right)\sigma_{3} }}{{\left( {E\varepsilon_{p} + 2\mu \sigma_{3} - \sigma_{r} } \right)\ln \frac{{\lambda \left( {E\varepsilon_{p} + 2\mu \sigma_{3} - \sigma_{r} } \right)}}{{\left( {\sigma_{p} - \sigma_{r} } \right)}}}} $$

14 $$ k = \left[ {\varepsilon_{p} - {{\left( {1 - 2\mu } \right)\sigma_{3} } \mathord{\left/ {\vphantom {{\left( {1 - 2\mu } \right)\sigma_{3} } E}} \right. \kern-0pt} E}} \right] \cdot \left[ {\frac{{m\left( {E\varepsilon_{p} + 2\mu \sigma_{3} - \sigma_{r} } \right)}}{{E\varepsilon_{p} - \left( {1 - 2\mu } \right)\sigma_{3} }}} \right]^{{m^{ - 1} }} $$

#### Determination of model parameters

Apply similar materials of mudstone and muddy limestone in Table 15 to the study of damage constitutive models for these two types of rocks. Obtaining $\lambda$, $E$, and $\mu$ (Poisson's ratio) of two types of rock similar materials through experiments. Then, the stress–strain curves under three different confining pressures after rainwater infiltration were obtained through triaxial tests, and the values of $\sigma_{P}$, $\varepsilon_{P}$ and $\sigma_{r}$ in Eq. (12) were determined. Finally, the model parameters $m$ and $k$ were obtained through Eqs. (13) and (14), as shown in Table 16.

**Table 16:** Relevant parameters of damage constitutive model.

Related parameters	$\lambda$	$E$(MPa)	$\mu$	$\sigma_{3}$(kPa)	$\sigma_{P}$(MPa)	$\varepsilon_{P}$(%)	$\sigma_{r}$(MPa)	Stage	$m$	$k$(× 10^–2^)
Mudstone	0.52	82.4	0.28	50	0.454	1.490	0.235	①	2.6417	2.1464
								②	1.3343	1.6031
				100	0.595	1.751	0.427	①	3.4573	2.4793
								②	1.0883	1.4366
				200	0.838	2.203	0.652	①	5.0092	2.9555
								②	1.0654	1.6734
Muddy limestone	0.59	152.02	0.26	100	1.030	1.221	0.607	①	10.6531	1.4927
								②	2.3316	1.4850
				200	1.322	1.553	0.801	①	9.6356	1.9014
								②	2.1486	1.8425
				300	1.623	1.920	1.025	①	8.1062	2.3930
								②	1.9221	2.1905

In this table, ① represents the deformation stage before the peak stress, and ② represents the stage after the peak stress.

#### Verification of damage constitutive model

**Figure 16:**
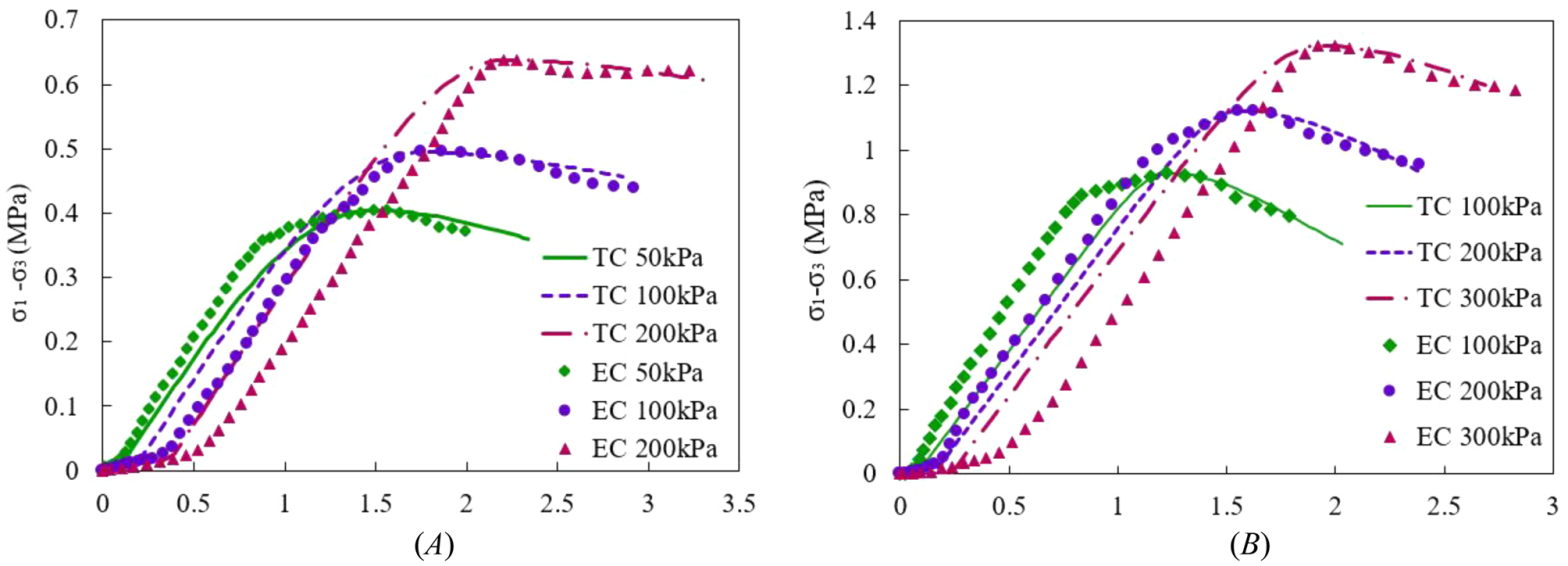
The principal stress–strain curves under various pressures (TC—Theoretical Curve; EC—Experiment Curve).

In order to further verify the applicability of the model, the experimental curves of similar materials of mudstone and muddy limestone were compared with the theoretical model curves, as shown in Fig. 16.

According to experimental and theoretical research, it has been found that the principal stress–strain curves of mudstone and muddy limestone after rainwater infiltration can be roughly divided into four stages: compaction stage, elastic stage, elastic–plastic stage, and plastic deformation stage. From the experimental results, it can be seen that there is no obvious compaction stage under low confining pressure, which is mainly attributed to the existence of pore water pressure. With the increase of confining pressure, the compaction stage becomes more obvious, and it is significantly longer than the theoretical results. In addition, the experimental values of the plastic deformation stage after the peak stress are close to the theoretical values. Under high confining pressure, the theoretical value of stress in the elastic stage is slightly greater than the experimental value, mainly due to the deviation caused by the existence of pores in the rock. Comparing the variation curves of mudstone and muddy limestone, it can be found that the higher the rock strength, the smaller the deviation between the theoretical and experimental values caused by pores. Overall, the impact of pores is not prominent and can be ignored, indicating the applicability of this model.

From the theoretical and experimental curves of the two types of rocks, it can be seen that after entering the plastic deformation stage, the decline rate of muddy limestone is faster than that of mudstone. It indicates that during the progressive failure process of these two types of rocks, the ability of mudstone damage elements to resist failure is stronger than that of muddy limestone, and it is more obvious under low confining pressure.

## Results and discussion

The article developed a similar material of rock with comprehensive parameters through orthogonal experiments, and combined regression analysis to prepare similar materials for mudstone and muddy limestone. On this basis, a further study was conducted on the damage constitutive model of rocks after rainwater infiltration, and its applicability was verified through triaxial tests. The above research findings:

1. Through orthogonal experiments, it was shown that ATC plays a dominant role in controlling the compressive strength, elastic modulus, cohesion, internal friction angle, and water absorption of similar materials, and these indexes show a significant decrease with the increase of ATC. For each level of ATC increase, the compressive strength decreases by 0.2 MPa, the elastic modulus decreases by 10–20 MPa, and the cohesion decreases by 25–45 kPa. With the ATC increases, the internal friction angle also decreases linearly. Gypsum can reduce strength and increase elastic modulus, with the former exhibiting significantly at low ATC and the latter exhibiting significantly at high ATC. In addition, with the increase of gypsum, the softening coefficient and permeability coefficient also significantly decrease. The addition of gypsum with a cement content of 4% -16% can meet the requirements of most sedimentary rock similar materials in terms of hydraulic properties. From this, it can be found that gypsum is an ideal material for regulating the hydraulic properties of similar materials in rocks.
2. Starting from studying the mechanical and hydraulic properties of similar materials, the experiment prepared a similar material with comprehensive parameters that is suitable for soft and hard rocks and fluid–solid coupling tests. This type of similar material can be used to simulate the progressive failure behavior of rock masses such as soft-hard interlayer and rainwater infiltration. The experimental results can also provide ideas for the study of the hydraulic properties of other types of similar materials. However, due to the use of gypsum and clay as raw materials, there are many pores in the material after coagulating and hardening. In addition, the test results of the most important parameter “*λ*” in the hydraulic property are 0.505–0.703. Therefore,this similar materials cannot be used to simulate hard, dense rocks.
3. Considering the effects of load and rainwater infiltration, the *Weibull* statistical damage theory is introduced to establish a statistical damage constitutive model for the triaxial compression process of rocks after rainwater infiltration. Triaxial tests were conducted on mudstone and muddy limestone under different confining pressures using similar materials that have been developed. The damage constitutive models of the two types of rocks after rainwater infiltration were verified. From the experimental results, it can be seen that there is no obvious compaction stage under low confining pressure, which is mainly attributed to the existence of pore water pressure. With the increase of confining pressure, the compaction stage becomes more obvious, and it is significantly longer than the theoretical results. In addition, the theoretical value of stress in the elastic stage under high confining pressure is slightly greater than the experimental value, but the higher the rock strength, the smaller the deviation between the theoretical value and the experimental value. Overall, the theoretical curve and experimental curve are basically consistent in terms of numerical values and variation trends, indicating that the model can be used to analyze the progressive failure behavior of rocks after rainfall infiltration.
4. After being eroded by rain, most of the soft rock show obvious changes in strength. The corresponding damage constitutive model is established by introducing the strength damage variable “*λ*” under the condition of rainwater infiltration. Therefore, this model is more suitable for simulating the damage process of soft rock under rainwater infiltration. However, due to the lack of consideration of joints, cracks and other factors, the application of this damage constitutive model has some limitations. In the following research, the authors will carry out a more in-depth discussion on the damage problems of these factors and rainwater infiltration coupling.

## Data Availability

The data from this study is available upon request from the corresponding author.
