# Author Correction: Development of similar materials for fluid–solid coupling model testing and application in damage constitutive models

**DOI:** 10.1038/s41598-024-69311-9

**Published:** 2024-08-09

**Authors:** Xu Ren, Guihong Xu, Ziwei Chen, Shixin Ran, Jie Zhang

**Affiliations:** 1 College of Civil Engineering, Guizhou Institute of Technology, Guiyang, 520025 China; 2 College of Civil Engineering, Guizhou University, Guiyang, 520025 China

Correction to: *Scientific Reports* 10.1038/s41598-024-65242-7, published online 26 June 2024

The Funding section in the original version of this Article was omitted. The Funding section now reads:

“This research was funded by the National Natural Science Foundation of China (51868009), Science and Technology Department Foundation of Guizhou Province (ZK-[2021] 296), Guiyang City Science and Technology Plan Project (ZHUKE HETONG [2024]-1-8), QIAN KE HE Platform Talents (GCC[2023]053) and The Key Laboratory of Chemical Admixture Science and Technology of Guizhou Province Higher Education Institutions (QJJ[2023]022).”

The original Article has been corrected.

